# Histopathological and ultrastructural analysis of vestibular endorgans in Meniere's disease reveals basement membrane pathology

**DOI:** 10.1186/1472-6815-9-4

**Published:** 2009-06-03

**Authors:** Andrew A McCall, Gail P Ishiyama, Ivan A Lopez, Sunita Bhuta, Steven Vetter, Akira Ishiyama

**Affiliations:** 1 Surgery Department, Division of Head and Neck "David Geffen" School of Medicine, at UCLA, Los Angeles, California, USA; 2 Neurology Department, "David Geffen" School of Medicine, at UCLA, Los Angeles, California, USA; 3 Department of Pathology, "David Geffen" School of Medicine, at UCLA, Los Angeles, California, USA

## Abstract

**Background:**

We report the systematic analysis of the ultrastructural and cytological histopathology of vestibular endorgans acquired from labyrinthectomy in Meniere's disease.

**Methods:**

17 subjects with intractable Meniere's disease and ipsilateral non-serviceable hearing presenting to the Neurotology Clinic from 1997 to 2006 who chose ablative labyrinthectomy (average age = 62 years; range 29–83 years) participated. The average duration of symptoms prior to surgery was 7 years (range 1–20 years).

**Results:**

Nearly all vestibular endorgans demonstrated varying degrees of degeneration. A monolayer of epithelial cells occurred significantly more frequently in the horizontal cristae (12/13 = 92%) (p < 0.001), the superior cristae (5/5 = 100%) (p < 0.005), the posterior cristae (2/2) compared with the utricular maculae (4/17 = 24%). Basement membrane (BM) thickening was more common in all of the cristae ampullares (18 out of 20) than the utricular maculae. Although only four saccular maculae were obtained, 3 out of 4 exhibited BM thickening and monolayer degeneration. Monolayer degeneration was highly significantly correlated with the presence of BM thickening (p < 0.001). Other degenerative changes noted equally among the five vestibular endorgans which were not significantly correlated with BM thickening or monolayer degeneration included hair cell vacuolization and stereocilia loss, microvesicles in the supporting cells, and increased stromal intercellular spaces. Transmission electron microscopy demonstrated disorganization of the BM collagen-like fibrils, and normal ultrastructural morphology of the nerve terminals and myelinated fibers. Stromal fibroblasts and endothelial cells of stromal blood vessels demonstrated vacuolization, and stromal perivascular BMs were also thickened.

**Conclusion:**

Systematic histopathological analysis of the vestibular endorgans from Meniere's disease demonstrated neuroepithelial degeneration which was highly correlated with an associated BM thickening. Other findings included hair cell and supporting cell microvessicles, increased intercellular clear spaces in the stroma, and endothelial cell vacuolization and stromal perivascular BM thickening.

## Background

In 1861, Prosper Ménière described the classical triad of episodic vertigo, fluctuating sensorineural hearing loss, tinnitus and a feeling of aural fullness, attributing the syndrome to an inner ear labyrinthine dysfunction [1]. Many theories on the pathophysiology of Meniere's disease have been postulated, including anatomic abnormalities affecting endolymph resorption, vascular abnormalities, post viral autoimmune mechanisms, and factors relating to water homeostasis [2-5]. However, the treatment of Meniere's disease is still based upon incomplete knowledge about the underlying pathophysiology. Thus, defining the ultrastructural and cytological histopathology of the vestibular endorgans is useful for developing a unifying theory of Meniere's disease.

The first temporal bone histopathological studies of Meniere's disease by Hallpike and Cairns [6] and Yamakawa [7] reported a ballooning distension of the endolymphatic system, a finding that is almost invariably seen in documented Meniere's disease [5,8]. Previous temporal bone studies had demonstrated evidence for endolymphatic membrane rupture or fibrosis around the endolymphatic sac. However, further temporal bone studies did not provide evidence for fibrosis of the endolymphatic sac [9]. Furthermore, primary and secondary endolymphatic hydrops has been documented in the temporal bones of subjects without symptoms of Meniere's disease [5], and thus hydrops is likely an epiphenomenon.

Previous histopathological studies of surgically obtained endorgans from Meniere's disease patients at the light microscopic level have posited a relative preservation of vestibular neuroepithelium [8,10,11] with variable degrees of ultrastructural changes under electron microscopy [12-16]. However, in many of the prior studies, only the utricular macula was systematically evaluated. We hypothesized differential degrees of histopathological changes would depend on the individual vestibular endorgan, and that ultrastructural and cytological abnormalities would correlate with the presence of neuroepithelial damage in the same endorgan, providing clues to the pathophysiology. Thus, we systematically evaluated the ultrastructural and histological features of the vestibular endorgans obtained from patients with intractable Meniere's disease undergoing transmastoid labyrinthectomy for treatment.

## Methods

### Patient Selection

The Institutional Review Board (IRB) of UCLA has approved this study. Appropriate informed consent was obtained from each patient before inclusion in the study. Patients who suffered from intractable vertigo spells despite medical management, and wished to undergo transmastoid labyrinthectomy were asked to participate voluntarily. All patients met the 1995 American Academy of Otolaryngology – Head and Neck Surgery (AAO-HNS) criteria for definite Meniere's disease [17]. All patients had non-serviceable hearing on the ipsilateral side. Patients with a history of previous ablative procedures (prior vestibular nerve section or transtympanic gentamicin application) were excluded. There were 17 patients (10 male, 7 female) from 1997 to 2005 who participated in the study. The demographic data, duration of symptoms prior to surgery, quantitative vestibular testing results are listed in Table 1. The average age was 62 years old (range 29–83 years old). The average duration of Meniere's disease symptoms prior to labyrinthectomy was seven years (range 1–20 years). All patients except one had varying degrees of ipsilateral caloric paresis.

**Table 1:** Clinical data of Meniere's disease patients at time of transmastoid labyrinthectomy.

Patient	Age	Sex	Disease Duration (years)	Side of Disease and Surgery	Vestibular/Caloric Testing
1	70	M	3.5	Right	Absent response on R
2	29	M	10	Left	90% L vestibular paresis
3	58	F	1	Left	81% L vestibular paresis
4	79	F	8	Left	36% L vestibular paresis
5	75	M	5	Left	35% L vestibular paresis
6	77	F	11	Left	25% L vestibular paresis
7	59	M	8	Right	60% R vestibular paresis
8	42	F	3	Left	90% L vestibular paresis
9	82	F	3	Right	Normal
10	83	M	3	Bilateral (surgery on left)	42% L vestibular paresis
11	56	M	1.5	Right	No record
12	34	F	1.5	Left	Incomplete testing^†^
13	54	M	20	Bilateral (surgery on left)	50% L vestibular paresis
14	32	M	9	Bilateral (surgery on left)	B vestibular dysfunction, L > R^‡^
15	65	M	1	Right	65% R vestibular paresis
16	81	F	19	Left	85% L vestibular paresis

17	56	M	3	Left	Non-specific, non-localized

†, vestibular testing incomplete secondary to patient intolerance; ‡, static positional nystagmus interfered with caloric measurements.

### Tissue processing

Individual vestibular endorgans obtained during transmastoid labyrinthectomy were received immediately in a mixture of 2% glutaraldehyde/4% paraformaldehyde (diluted in sodium phosphate buffer, 0.11 M, pH 7.4) and post-fixed for 16 hours. Vestibular endorgans were then immersed in 1% osmium tetroxide diluted in phosphate buffer solution (PBS) for one hour and were subsequently washed with PBS. The endorgans were dehydrated with ascending alcohols (70–95% in distilled water), 100% ethanol, 100% propylene oxide, and then embedded in EPON-araldite (EMS). Two-micron thick serial sections were obtained using a Micron Ultramicrotome HM355 S. Sections were counterstained with 1% toluidine blue solution and then cover-slipped with permanent mounting media (Permount, Fisher Scientific). Tissue sections were viewed and imaged in a Nikon Eclipse E800 fluorescent microscope equipped with RTSlider spot digital camera and Image Pro PlusTM software. Images were processed using the Adobe Photoshop software program run on a Macintosh iMAC computer.

For transmission electron microscopy (TEM) examination, ultrathin (70 nm) sections were made (from the same specimens used for light microscopic examination) with a diamond knife (Diatome/Polysciences) on a Richter III ultramicrotome. Sections were mounted on single slot Formvar-coated copper grids (EMS) and counterstained with uranyl acetate (30 minutes) and lead citrate (5 minutes), before observation with a Jeol 100 CX (Osaka, Japan). All sections were studied at low (×4500) and higher magnification view (×15000).

### Data acquisition and analysis

In all cases, the utricular maculae were successfully harvested and processed. Four saccular macula, 20 cristae ampullares (13 horizontal, five superior, and two posterior) were harvested and processed. Technical aspects of transmastoid labyrinthectomy precluded the successful harvesting and preservation of some of the endorgans, most often as the result of crush artifact of the smaller vestibular endorgans. Endorgans were examined by light microscopy and TEM.

The normal cytoarchitecture of the human vestibular sensory epithelium has been described [18-20]. Type I hair cells are characterized by their flask-shape, circular nuclei, and a surrounding goblet-shaped nerve calyx. Type II hair cells have cylindrical shape and oval nuclei. Both type I and II hair cells have stereocilia at their apical surface. Supporting cell nuclei lie at the basal surface of the epithelium, have irregular shape, and stain intensely. Tissue specimens were analyzed for the presence or absence of type I and type II hair cells in the sensory epithelia, loss of stereocilia from hair cells, vacuolization of air cells and supporting cells, and the presence or absence of calyceal terminals in the sensory epithelia and monolayer transformation of the sensory epithelium. The basement membrane (BM) is a continuous network of extracellular proteins and proteoglycans located at the epithelial and mesenchymal interface of most tissues, approximately 40–100 nm thick [21]. We have recently described the composition and immunolocalization of the human inner ear basement membranes [22]. The stroma underneath the sensory epithelia was analyzed for changes that include BM thickening, the presence and appearance of nerve fibers, and increased intercellular stromal spaces.

BM measurements: BM thickness was measured using the Microsuite™ Five (MS5) software run on a Dell Precision 380 computer, coupled to a light and fluorescent microscope Olympus BX51. Images were captured using a CCD camera DP70 attached to the microscope. Toluidine blue counterstained cross-sections of the utricular maculae (n = 9), saccular maculae (n = 3), horizontal (n = 11), superior and posterior (n = 2) cristae ampullares were observed and captured at 60×. For each endorgan, the BM was identified below the sensory epithelia. Using the command "Vertical line" from the MS5 software, five measurements were made at a set measured distance along the sensory epithelia cross-section. The measurements were averaged (n = 5) and the standard error of the mean was determined using SIGMA-Stat software. All other statistical analysis of the data obtained was performed on SAS software utilizing Fisher's exact test.

## Results

### Neuroepithelial degeneration

**Figure 1:**
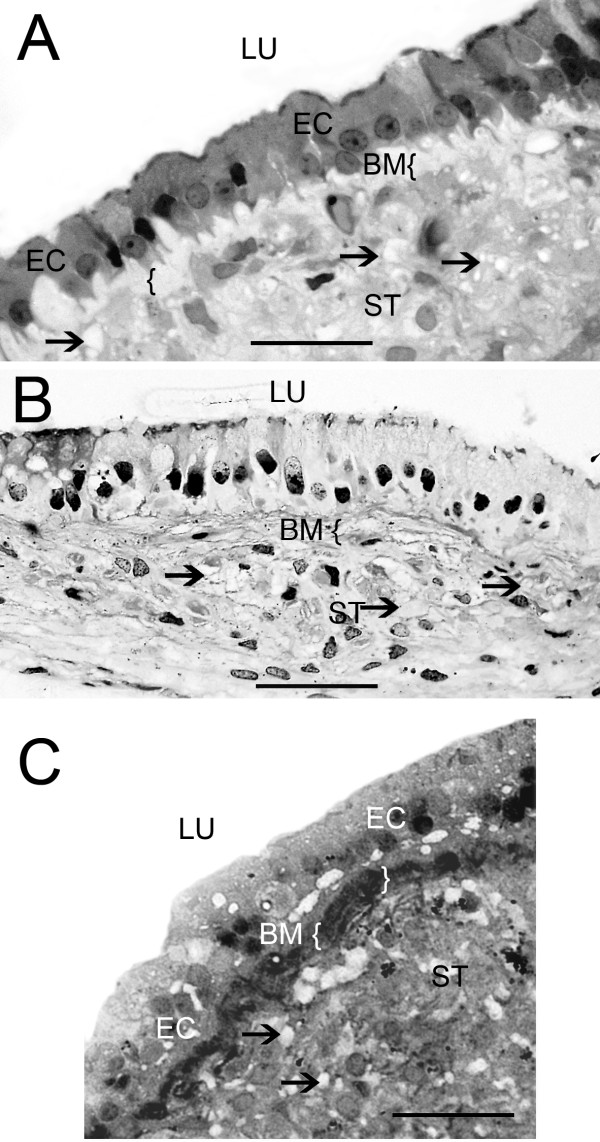
**Light microscopic photomicrograph of the sensory epithelia from Meniere's disease vestibular endorgans**. (A) Cross-section of the horizontal crista from a subject with Meniere's disease. The sensory epithelia (SE) demonstrated degeneration and conversion from the normal cytoarchitecture to a single layer of epithelial cells (EC) with a columnar shape, uniform-appearing cytoplasm, and homogeneously-staining nuclei, aligned at the same level between the apical and basal surfaces of the cell. The basement membrane (BM) was severely thickened ({ }). The stroma (ST) demonstrated mild vacuolization with increased intercellular spaces (arrows). (B) Cross-section of a utricular macula from another subject with Meniere's disease. The hair cells (HCs) and supporting cells (SCs) formed almost a single layer. There was also thickening of the underlying BM. (C) Cross-section of a posterior crista from another subject with Meniere's disease. The SE is devoid of HCs and the remaining SCs form a monolayer. There was a pronounced thickening of the BM ({ }). Bar A, B and C = 25 μm. Two-micron thin sections counterstained with osmium tetroxide and toluidine blue.

There were varied degrees of neuroepithelial degeneration with more severe degeneration of the semicircular canal cristae ampullares and saccular maculae, than of the utricular maculae. Figures 1A and 1B illustrate a frequently observed pattern of epithelial degeneration: a monolayer of columnar shaped epithelial cells with a uniform-appearing cytoplasm, homogeneously-staining nuclei aligned at the same level between the apical and basal surfaces of the cell. When present, the epithelial monolayer replaced a variable amount of the normal vestibular sensory epithelium, ranging from partial to nearly complete (Fig. 1A, Table 2). There was a significantly higher frequency of this histopathological finding in the horizontal cristae (12 out of 13; p < 0.001) and in the superior cristae (5 out of 5; p < 0.005) compared with the utricular macula (4 out of 17). Observations from the posterior semicircular canals from two patients showed similar degenerative changes i.e. monolayer epithelialization, stromal edema and BM thickening (Fig. 1C). The proportion of specimens demonstrating epithelial monolayer transformation of the utricular maculae compared with the saccular maculae (3 out of 4) trended toward statistical significance (p = 0.09). Of note, the saccular maculae were oftentimes visibly atrophic under the operative microscope, and thus difficult to obtain and many of these would likely have demonstrated degenerative changes.

**Table 2:** Epithelial Monolayer change in vestibular endorgans harvested from patients with Meniere's disease.

Patient Number	Utricle	Saccule	HSCC	SSCC
1	N	Y	Y	-
2	Y	Y	Y	-
3	N	-	Y	-
4	N	-	-	Y
5	N	-	Y	Y
6	Y	-	Y	-
7	N	Y	Y	Y
8	N	-	Y	-
9	N	-	Y	-
10	N	-	Y	-
11	N	-	N	-
12	Y	-	-	-
13	N	-	Y	-
14	Y	-	Y	Y
15	N	-	-	-
16	N	N	Y	Y
17	N	-	-	-

% with Epithelial Monolayer	24%^†^	75%	92%	100%

HSCC, horizontal semicircular canal crista ampullaris; SSCC, superior semicircular canal crista ampullaris; Y, presence of epithelial monolayer change detected; N, absence of epithelial monolayer change, - = tissue non-available.

† = Statistically significant difference in proportion of epithelial monolayer change of utricular macula compared with horizontal semicircular canal crista ampullaris (p < 0.001) and superior semicircular canal crista ampullaris (p = 0.005).

### Thickening of the BM beneath the epithelium

**Figure 2:**
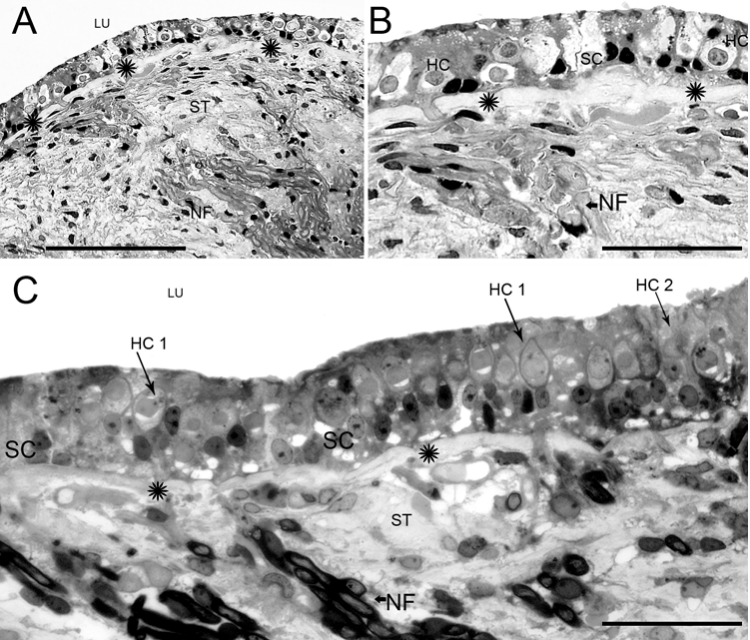
**Light microscopy photomicrograph of the superior crista and utricular macula from patients with Meniere's disease**. (A) Cross-section of the superior crista at the planum semilunatum level. The SE demonstrated disorganization of the cytoarchitecture. Stereocilia were absent from the lumen (LU). The underlying stroma (ST) appeared normal. (B) High magnification view from (A). The epithelium demonstrated HC (HC) loss and degenerative changes. HCs showed perinuclear vacuoles and SC (SC) cytoplasm was expanded into areas of HC loss. The stroma, fibroblast and nerve fibers (NF) appeared normal. Thickening of the BM underneath the SE was prominent (asterisk). (C) Cross-section of the macula utricle from another patient with Meniere's disease. The SE was almost normal. Type I HCs (HC 1) with their calyces are easily identified. There were no stereocilia present. Type II HCs were also noted (HC 2). The u BM demonstrated mild thickening (asterisk). The ST and NF within are normal. Bar in A = 125 μm, B = 50 μm, C = 40 μm. Two-micron thin sections counterstained with osmium tetroxide and toluidine blue.

A uniform thickening of the BM underlying the vestibular neuroepithelium was frequently observed throughout the affected endorgan (Fig. 1C, 2A and 2B). All endorgans with epithelial monolayer degeneration had an associated thickening of the underlying BM, and focal areas of epithelial degeneration were often associated with underlying BM thickening (Fig. 2A and 2B). The presence of monolayer degeneration was significantly correlated with the presence of BM thickening (p < 0.001). BM measurements of affected endorgans are shown in Table 3. BM thickening was noted in 18 out of 20 of the cristae ampullares, and 3 out of 4 of the saccular maculae, but only 9 out of 17 of the utricular maculae (Table 3). The BM thickness of the utricular maculae ranged from 1.87 to 2.86 μm, of the saccular maculae, it ranged from 3.03 to 3.42 μm, of the horizontal cristae, it ranged from 2.01 to 4.74 μm and of the superior cristae, and it ranged from 2.04 to 3.79 μm. The BM thickness of the horizontal cristae (n = 11) was significantly greater than that of the utricular maculae (n = 9) using a Mann-Whitney Rank sum test (p < 0.001). In specimens from Meniere's disease which were designated as not thickened, the BM was not discernible or measured less than 0.5 μm as in the normal utricular maculae, saccular maculae and cristae BM obtained from autopsy (not shown).

**Table 3:** Basement membrane thickening in vestibular endorgans harvested from patients with Meniere's disease.

Patient Number	Utricle	Saccule	HSCC	SSCC
1	N	Y(3.03 ± 0.15)	Y(3.34 ± 0.1)	-
2	Y(2.86 ± 0.09)	Y(2.08 ± 0.07)	Y(2.26 ± 0.09)	-
3	N	-	Y(2.2 ± 0.1)	-
4	Y(2.79 ± 0.09)	-	-	Y(2.74 ± 0.23)
5	N	-	Y(3.97 ± 0.18)	Y(3.79 ± 0.1)
6	Y(2.01 ± 01)	-	Y(4.74 ± 0.17)	-
7	N	Y(3.42 ± 0.11)	Y(3.75 ± 0.28)	Y(2.88 ± 0.12)
8	Y(2.2 ± 0.08)	-	Y(4 ± 0.19)	-
9	Y(2.63 ± 0.34)	-	Y(2.6 ± 0.5)	-
10	N	-	Y(3.38 ± 0.15)	-
11	N	-	N	-
12	Y(2.32 ± 0.2)	-	-	-
13	Y(2.67 ± 0.20	-	N	-
14	Y(1.9 ± 0.28)	-	Y(2.01 ± 0.07)	Y(2.04 ± 0.1)
15	Y(1.87 ± 0.05)	-	-	-
16	N	N	Y(2.74 ± 0.23)	Y(2.74 ± 0.12)
17	N	-	-	-

%of tissue with BM thickening	53%	75%	85%	100%

HSCC, horizontal semicircular canal crista ampullaris; SSCC, superior semicircular canal crista ampullaris; Y: yes, BM thickening detected; ± = standard error of the mean from 5 measurements in a representative cross section. N= no, absence or faintly discernible BM thickening. In these cases, BM thickness was less than 0.5 micron, tissue non available.

### Other histopathological changes

Other vestibular epithelial changes were non-specific, occurring relatively equally in all of the vestibular endorgans (Tables 4 and 5). These included loss of hair cell stereocilia (range 75–100%) and vacuolization of the hair cells and supporting cells (range 69–82%) (Table 4). Type I and type II hair cells frequently exhibited perinuclear vacuolization, a feature noted with relative equal frequency in all endorgans (Table 5), and there was no apparent differential degree of degeneration dependent on type I vs. type II hair cell classification. In most of the specimens, calyces and stromal nerve fibers appeared normal without axoplasmic vacuolization or myelin disorganization (Fig. 2B and 2C).

**Table 4:** Loss of hair cell stereocilia in vestibular endorgans harvested from patients with Meniere's disease.

Patient Number	Utricle	Saccule	HSCC	SSCC
1	Y	Y	Y	-
2	Y	Y	Y	-
3	Y	-	Y	-
4	Y	-	-	Y
5	Y	-	Y	Y
6	Y	-	Y	-
7	Y	Y	Y	Y
8	Y	-	Y	-
9	Y	-	Y	-
10	Y	-	Y	-
11	Y	-	Y	-
12	Y	-	-	-
13	Y	-	Y	-
14	Y	-	Y	Y
15	Y	-	-	-
16	N	N	Y	N
17	Y	-	-	-

% with loss of stereocilia	94%	75%	100%	80%

HSCC, horizontal semicircular canal crista ampullaris; SSCC, superior semicircular canal crista ampullaris; Y, loss of hair cell stereocilia; N, normal stereocilia noted, - = tissue non-available.

**Table 5:** Cellular vacuolization of epithelial cells in vestibular endorgans harvested from patients with Meniere's disease.

Patient Number	Utricle	Saccule	HSCC	SSCC
1	N	Y	Y	-
2	Y	Y	Y	-
3	Y	-	Y	-
4	Y	-	-	N
5	Y	-	Y	Y
6	Y	-	N	-
7	Y	Y	Y	Y
8	Y	-	N	-
9	Y	-	Y	-
10	Y	-	Y	-
11	Y	-	N	-
12	Y	-	-	-
13	Y	-	N	-
14	Y	-	Y	Y
15	Y	-	-	-
16	N	N	Y	Y
17	N	-	-	-

% with cellular vacuolization	82%	75%	69%	80%

HSCC, horizontal semicircular canal crista ampullaris; SSCC, superior semicircular canal crista ampullaris; Y, presence of epithelial cellular vacuolization detected; N, absence of epithelial cellular vacuolization, - = tissue non-available.

### Stromal fibrocytes

**Figure 3:**
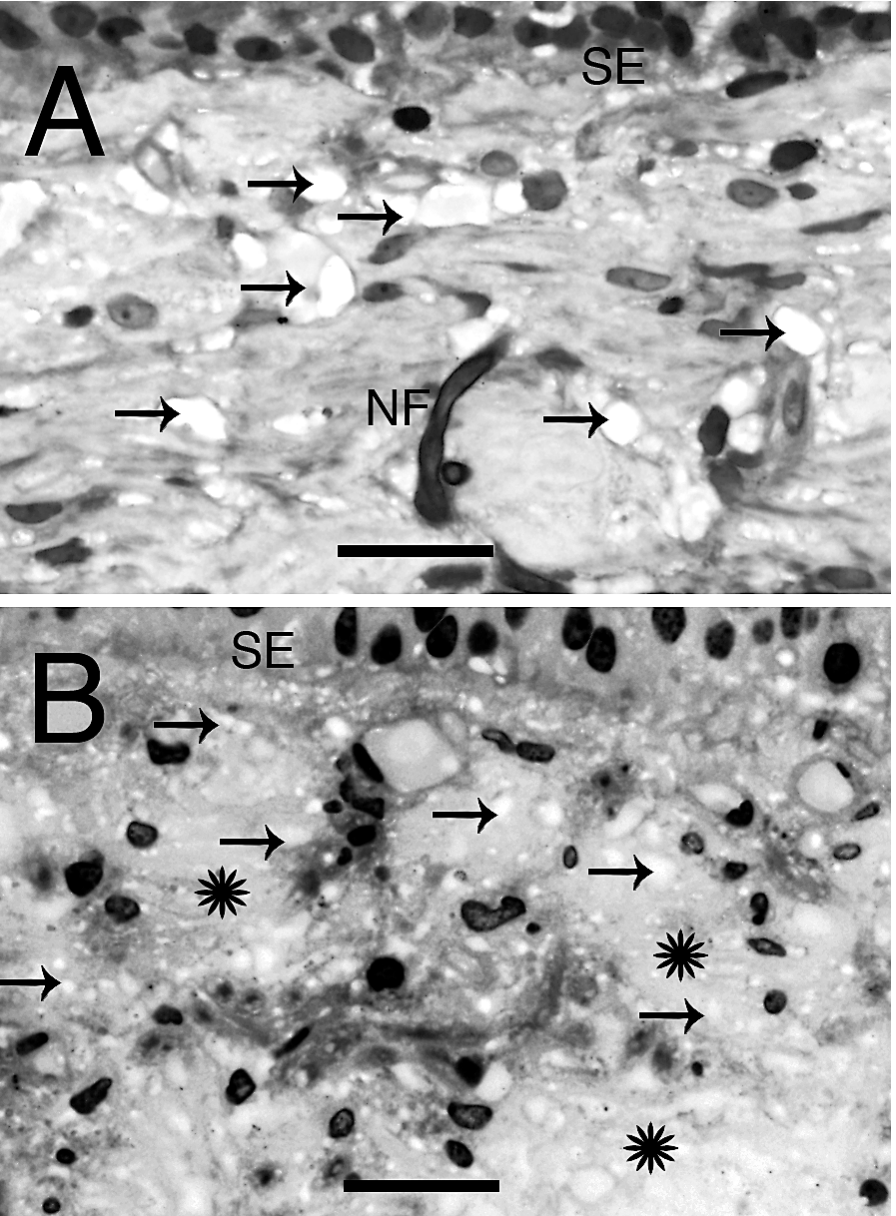
**Light microscopy photomicrographs of the stroma underneath the sensory epithelia (SE) of patients with Meniere's disease**. (A) Empty spaces within the stroma, resembling vacuolization or interstitial edema, were seen in the utricular stroma from Meniere's vestibular endorgans (arrows). NF showed almost normal appearance (NF). (B) The utricular stroma from another patient with Meniere's disease showing intercellular spaces (asterisks) and pronounced vacuolar structures (arrows). Bar in A and B = 35 μm. Two-micron thin sections counterstained with osmium tetroxide and toluidine blue.

In most of the specimens, there appeared to be more numerous fibrocytes with an apparent alteration of their morphology from a more rounded shape to an elongated and flattened shape. Frequently the fibrocytes were aligned with the BM underlying the epithelium (Fig. 2B). In some cases, the cytoplasm of the fibrocytes was vacuolated, and increased intercellular empty spaces resembling interstitial edema were present in the stroma surrounding the fibrocytes (Fig. 3A and 3B). Subepithelial stromal tissue increased interstitial empty spaces or edema was a frequent finding that ranged from 50% of saccular maculae specimens to 80% of superior semicircular canal cristae ampullares specimens (Table 6).

**Table 6:** Increased intercellular stromal spaces in vestibular endorgans harvested from patients with Meniere's disease.

Patient Number	Utricle	Saccule	HSCC	SSCC
1	N	N	Y	-
2	Y	Y	N	-
3	Y	-	Y	-
4	N	-	-	N
5	Y	-	Y	Y
6	Y	-	Y	-
7	Y	Y	Y	Y
8	Y	-	Y	-
9	Y	-	Y	-
10	Y	-	Y	-
11	Y	-	N	-
12	Y	-	-	-
13	Y	-	N	-
14	Y	-	Y	Y
15	Y	-	-	-
16	N	N	N	Y
17	N	-	-	-

Percent with increased stromal spaces	76%	50%	69%	80%

HSCC, horizontal semicircular canal crista ampullaris; SSCC, superior semicircular canal crista ampullaris; Y, presence of increased intercellular stromal spaces detected; N, normal intercellular stromal spaces.

### Comparison of histopathological findings within subjects

There was a strong correlation between the presence of neuroepithelial monolayer degenerative changes and the presence of thickening of the underlying BM (p < 0.001). Of endorgans exhibiting degeneration to a monolayer (4 utricular, 3 saccular, 13 horizontal cristae, 5 superior cristae and 2 posterior crista), all cases with the exception of one (patient # 13) were associated with BM thickening. However, there were multiple cases of thickening of the underlying BM without conversion to a monolayer, particularly in the utricular maculae (patients 4, 8, 9, 13 and 15). There appeared to be a spectrum of epithelial damage as virtually all endorgans, including the utricular maculae, exhibited hair cell loss and drop-out. The finding of intercellular stromal spaces did not appear to be correlated with either BM thickening or epithelial monolayer degeneration, as this finding was noted in utricular maculae without BM thickening or monolayer degeneration (patients 3, 5, 7, 8, 9, 19, 11, 13, 15) and was not found in horizontal and superior cristae that had BM thickening and monolayer (patients 3, 13, 16 horizontal and patient 4 superior cristae).

### Ultrastructural findings under transmission electron microscopy (TEM)

**Figure 4:**
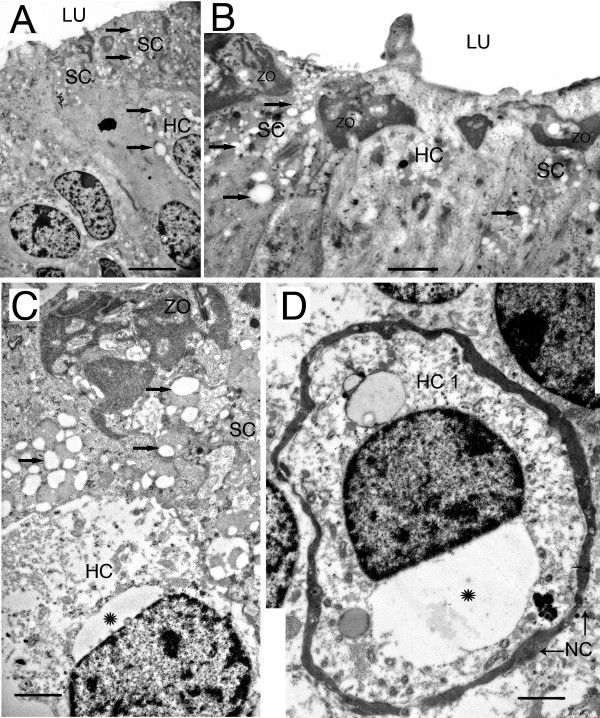
**Transmission electron micrographs of the sensory epithelia from patients with Meniere's disease**. (A) The luminal portion (LU) of the crista SE shows no stereocilia and no stereocilia roots. Vacuolization of HCs (HC) and the apical portion of supporting cell (SC) cytoplasm was frequently observed (short arrows). (B) Higher magnification portion of the luminal portion of the SC demonstrates vacuoles (short arrows). The zonula occludens (ZO) region, where tight junctions are located, appeared disorganized with numerous vacuoles (ZO). (C) The apical portion of the utricular macula epithelia. There was a pronounced vacuolization or microvessicles of the upper cytoplasm of SC (short arrows). The HC often demonstrated perinuclear vacuoles (asterisk). (D) A type I HC (HC 1) is easily identifiable by their surrounding nerve calyx (NC). Note the perinuclear vacuolization (asterisk). Bar in A = 5 μm, B = 2 μm, C = 1 μm, D = 1.5 μm. Ultrathin sections counterstained with uranyl acetate and lead citrate.

Under TEM, there were disorganized collagen-like fibrils in epithelial and perivascular BMs, vacuolization of fibrocytes and endothelial cells. Transmission electron microscopy observations were also significant for small vesicle formations in the apical portion of supporting cells (Fig. 4A, B and 4C). In most of the hair cells, the stereocilia were missing with an absence of stereocilia roots (Fig. 4A). The region of the zonula occludens, at the apical portion of the sensory epithelia where tight junctions are located, appeared to be disorganized (Fig. 4B). Type I hair cells showed the characteristic perinuclear vacuoles (Fig. 4C and 4D). The nerve calyces surrounding type I hair cells appeared to be well-preserved (Fig. 4D).

**Figure 5:**
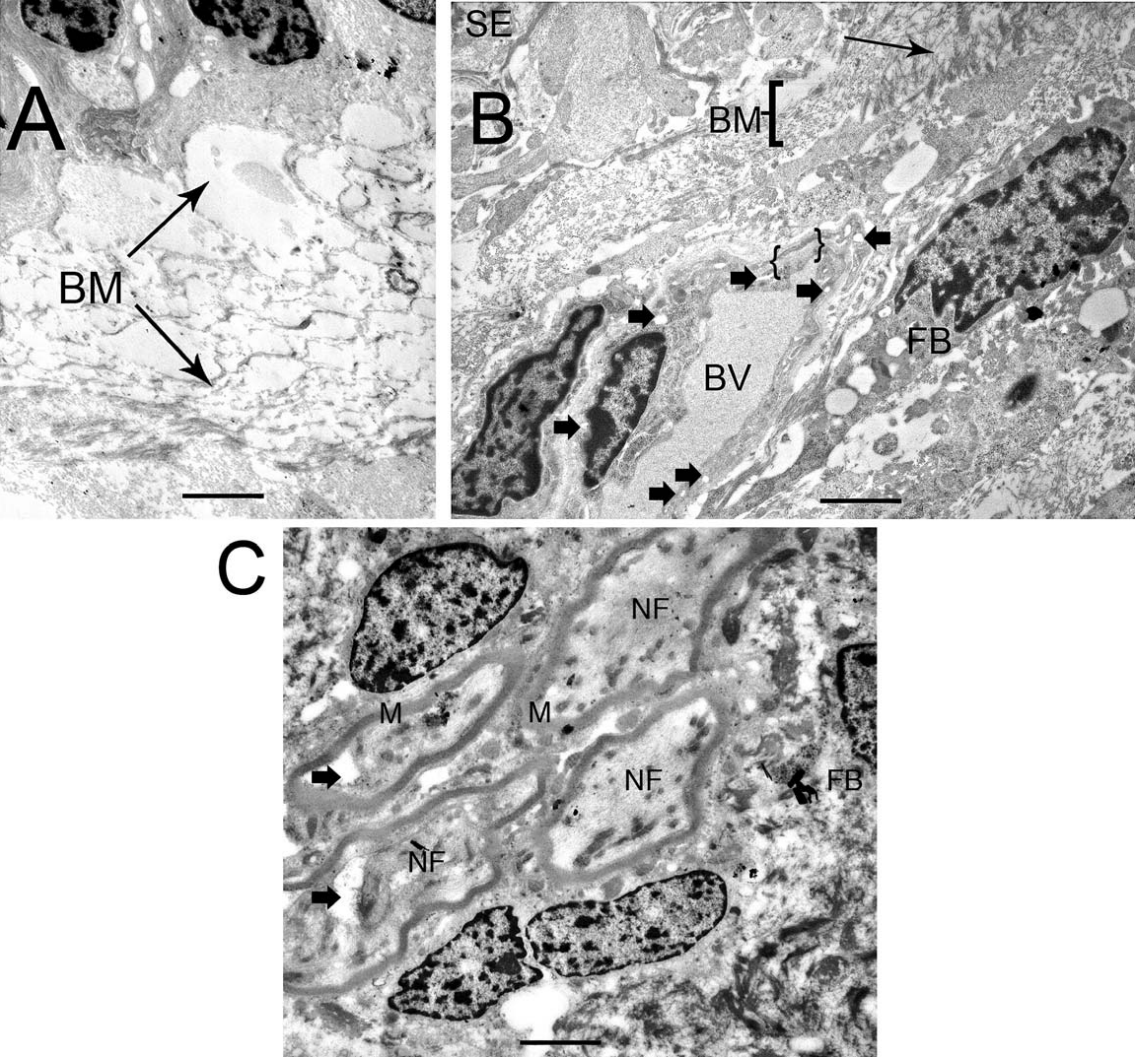
**Transmission electron micrographs of the basement membrane and stroma beneath the sensory epithelia from patients with Meniere's disease**. (A) Horizontal crista ampullaris from a patient with Meniere's disease. The thickening of the BM underneath the SE appeared to be disorganized collagen-like fibrils under TEM. (B) BM from a Meniere's utricular macula. The thickening of the BM appeared to be a disarray of collagen-like fibrils (arrows). The stromal fibroblast (FB) cytoplasm demonstrated mild vacuolization. The stromal perivascular BMs surrounding the blood vessels (BV) was also thickened ({ }), and endothelial cell cytoplasm was vacuolated (arrowheads). (C) Nerve fibers (NF) within the stroma appear relatively normal. The NF axoplasm is mildly vacuolated (arrowheads), with normal myelin arrangement (M). Bars in A = 6 μm, B = 5 μm, C = 6 μm. Thin sections counterstained with uranyl acetate and lead citrate.

The BM was thickened underneath the sensory epithelia, and electron microscopy revealed disorganized collagen-like bundles (Fig. 5A and 5B). Within the underlying stroma, fibroblasts exhibited vacuolization (Fig. 5B). The perivascular BMs demonstrated thickening, and endothelial cell cytoplasm contained numerous vacuoles (Fig 5B). There were fibrillary depositions close to the blood vessels and fibrocytes of the stroma. There were no cellular infiltrates or evidence of inflammatory changes. The nerve fiber axoplasm showed mild vacuolization with preservation of the surrounding myelin sheaths (Fig. 5C).

### Clinicopathological Correlations

Nearly all subjects with monolayer formation had significant caloric paresis. As noted prior, the presence of monolayer formation was highly significantly correlated with BM thickening, potentially indicative of a related pathology. However, the degree of BM thickening in the horizontal crista ampullaris was not correlated with the degree of caloric paresis and the degree of BM thickening was also not correlated with length of disease prior to surgery.

## Discussion

### Analysis of histopathological features of the vestibular sensory epithelium in Meniere's disease and clinical implications

It is noteworthy that despite the heterogeneity of ages of the patients (ages ranging from 29 to 83 years of age) and the length of time with symptoms of Meniere's disease prior to surgery (ranging from 1 to 20 years), there was a remarkably similarity of the endorgan histopathology between all subjects. This is suggestive that there may be a final common pathophysiological pathway for intractable stage IV Meniere's disease.

A common pattern of degeneration was partial or complete replacement of the normal epithelial cytoarchitecture by a monolayer of cells with a differential frequency of occurrence: 92% of the horizontal cristae and 75% of the saccular macula, compared with 24% of the utricular macula. These findings contrast with prior studies reporting normal cytoarchitecture of the vestibular sensory epithelium under light microscopy in Meniere's disease [11,16,23]. A potential explanation is the relative infrequency of epithelial monolayer degeneration in the utricular macula, although we cannot exclude the possibility of more severe disease in our patients. Given the difficulties inherent in harvesting and preserving other endorgans, most prior studies have systematically analyzed only the utricular maculae [12-14,16]. Ylikoski et al. [11] reported surgical acquisition of the utricular macula and horizontal canal cristae from 11 patients with Meniere's disease; although, it is not clear how many canal cristae ampullares were successfully harvested and analyzed. The present study represents the first systematic analysis of endorgan specimens from a large number of patients with intractable Meniere's disease, and is the first to document histopathological differences dependent on the endorgan.

Horner [24] analyzed the post-mortem utricular and saccular macula from one patient with Meniere's disease with a history of vestibular nerve section ten years prior. In that case study, the saccular macula exhibited severe degenerative changes with only a few normal appearing sensory cells, and a denuding of ciliary tufts. The utricular macula of the same patient was relatively spared. We also noted relative sparing of the utricle, and in our study since we excluded patients with a history of prior surgery, there is no confounding effect of prior surgical or chemical denervation.

With regard to clinical implications, the consistent finding of monolayer degeneration of the horizontal cristae ampullares in intractable Meniere's disease is the likely explanation for the frequent occurrence of caloric paresis. Notably, the presence of monolayer degeneration was significantly associated with the presence of caloric paresis (p < 0.001), and monolayer formation was significantly associated with BM thickening (p < 0.001). There was not a significant correlation between the degree of BM thickening and the degree of caloric paresis. This is likely secondary to individual differences of the effect of hair cell loss on the degree of caloric paresis, or the differential effect of BM thickening on epithelial changes. Unfortunately, vestibular evoked myogenic potentials (VEMPs), which reflect saccular function, were not available at the time that these specimens were acquired.

The relative sparing of the utricle to degenerative changes in Meniere's disease warrants further investigation. The utricular macula was relatively spared in age-related hair cell loss compared with the cristae ampullares [25,26] and our laboratory applied unbiased stereology to demonstrate an age-related loss of type I and type II hair cells in the human horizontal cristae [19], and in a subset of the same subjects, a lack of an age-related decline in type I or type II hair cell counts in the utricular macula [18]. The utricular macula was also relatively spared in intratympanic gentamicin for intractable Meniere's disease [27]. There are likely biochemical, anatomical, and vascular differences between the endorgans as the human horizontal crista ampullaris has a type I: type II ratio of near unity [19], and the human utricular macula has ratio of 1.7 [18]. Tsuji et al. [28] reported a significant reduction of type II hair cell density, but not a significant reduction of type I hair cell density in Meniere's disease. In the present study, there appeared to be a relatively equal loss of type I and type II hair cells in endorgans which exhibited hair cell loss. However, type I and type II hair cells cannot be discerned in the endorgans with monolayer degeneration. The process may initially begin with hair cell loss and loss of BM homeostasis and then progress to an epithelial monolayer with severe BM thickening.

Other sensory epithelial changes, including the loss and shortening of hair cell stereocilia and cellular vacuolization, were observed equally frequently among the vestibular endorgans. These non-specific changes have been reported in prior studies, which for the most part studied the utricular maculae [11,13,14,16]. Our study demonstrates that these findings occur amongst all of the individual vestibular endorgans. Loss of hair cell stereocilia may be directly related to Meniere's disease pathology, surgical manipulation or tissue processing, as stereocilial loss has been reported in surgically acquired acoustic neuroma specimens; however, in acoustic neuroma specimens the remaining stereocilial roots were noted [29]. In our specimens, stereocilial roots were not present. In studies of the endolymphatic sac from patients undergoing surgical resection, stereocilia-like structures have been found in the all of the endolymphatic sacs of surgically acquired specimens for Meniere's disease, but were found in only one out of 18 specimens from acoustic neuroma [30], similarly to animal models [31]. Stereocilial loss has been reported in aging as well [14]. In our study, increasing age was not correlated with loss of stereocilia, and the one patient whose endorgans exhibited stereocilia was 81 years old (patient #16); the two younger patients (29 and 32 years old) had stereocilia loss.

In the present study, supporting cell vesicles and hair cell vacuoles were observed in the majority of all endorgans from subjects with Meniere's disease. Previous histopathological studies of surgically acquired vestibular endorgans from Meniere's disease have noted vesicle formation within the supporting cell cytoplasm, particularly in the apical and middle regions, described as a "foamy appearance of the cell" [11,12,32]. Vacuoles have also been reported in both vestibular hair cells and supporting cells in acoustic neuroma specimens, more numerous in aging, without correlation with tumor size or caloric paresis [29]. Sensory hair cell and supporting cell vacuoles have also been reported in Meniere's specimens, with reports of increased vacuolization with aging [13,14,16].

### Analysis of histopathological features of the basement membrane and stroma underlying the vestibular sensory epithelium in Meniere's disease

A consistently observed striking histopathology was thickening of the BM underlying the sensory and non-sensory epithelium, a finding which was highly significantly correlated with epithelial degeneration. In a total of 24 endorgans which exhibited monolayer degeneration, only one endorgan did not have an associated BM thickening. However, not all endorgans exhibiting BM thickening had degeneration to a monolayer. Neuroepithelial BM thickening occurred less frequently in the utricular macula which also exhibited less frequently epithelial degeneration to a monolayer. Basal lamina thickening of the non-sensory utricle and endolymphatic duct was noted in surgical specimens from a patient with Meniere's disease [33]. However, their study did not comment on BM pathology underlying the sensory portion of the vestibular endorgans. One of the reasons that prior studies may not have observed BM thickening is that human temporal bones were traditionally embedded in celloidin and sectioned at 20 microns. Likely because of the thickness of the sections or the difficulty to visualize the BM under hematoxylin and eosin, fine alterations such as BM thickening had not previously been described.

Renal BM thickening may occur after a preceding vascular event with subsequent scarring, secondary to a form of deposition disease, or as a reparative process [34]. A focal thickening of the BM, increased deposition of collagen fibers, and lymphoid infiltrates in the endolymphatic sac epithelium from a patient with Meniere's disease has been reported, however prior ultrasound treatment may have caused an altered cellular response [35]. Immunological challenge to the endolymphatic sac produces endolymphatic hydrops and cochleo-vestibular disturbances in animal models [36,37]. However, in all of the endorgans in the present study, there were no lymphoid cells or inflammatory changes. In the kidney, the BM plays a critical role in solute and ion transport regulation [38], and thus the thickened BM in the vestibular endorgan may exhibit dysfunctional regulation of endolymphatic fluid and ionic composition.

In the majority of the endorgans, the vestibular stroma underlying the sensory epithelium was notable for increased intercellular stromal spaces. However, the increased intercellular spaces did not correlate with either BM thickening or epithelial degeneration to a monolayer. Surgical manipulation or tissue processing artifact cannot be ruled out. In the present study, ultrastructural pathological vacuolization was noted in the stromal fibrocytes in endorgans from Meniere's patients. Fibrocytic pathology in the spiral ligament of the aged gerbil has been studied extensively [39]. The spiral ligament contains specialized fibrocytes, and both the spiral ligament and the stria vascularis exhibited volume loss in Meniere's disease compared with age-matched normatives [40], indicative of spiral ligamental pathology in Meniere's disease. In agreement with a previous study of the utricular maculae from patients with Meniere's disease [11], stromal nerve fibers appeared to be normal in all endorgans. The endothelial cells of the stromal blood vessels also exhibited vacuolization, which may indicate microvascular pathology in the vestibular endorgans in Meniere's disease.

## Conclusion

The power of the present study was the ability to study patterns of histopathological alterations both between subjects and within subjects by investigating each individual endorgan. The majority of the vestibular endorgans acquired during surgical ablation from patients with intractable Meniere's disease demonstrated variable degrees of neuroepithelial degeneration including conversion of the sensory epithelium to a monolayer, BM thickening, cellular vacuolization, absence of hair cell stereocilia, and increased intercellular stromal spaces. The utricular macula was relatively spared, with a significantly less frequent finding of epithelial degeneration and BM thickening. Other findings such as loss of stereocilia and cellular vacuolization were observed equally amongst the endorgan types. Under transmission electron microscopy, there were disorganized collagen-like fibrils near BMs, disorganization of the zona occludens, and apparent alterations in stromal fibrocyte and vascular endothelial cell morphology. Further studies are indicated to evaluate the role of the BMs in normal vestibular physiology and in Meniere's disease.

## Competing interests

The authors declare that they have no competing interests.

## Authors' contributions

AM wrote manuscript draft, performed quantitative analysis, collected surgical specimens, perform evaluation of morphology. GI edited manuscript, designed and coordinated quantitative analysis, and developed methodology. AM and GI contributed equally to this manuscript. IAL performed staining and microscopic analysis, prepared and analyzed histological samples, and prepared micrographs. SB supervised transmission electron microscopy observations. SV carried out transmission electron microscopy AI Obtain all surgical specimens, and designed and conceived the study. All authors read and approved the final manuscript.

## Pre-publication history

The pre-publication history for this paper can be accessed here:


